# White noise speech illusions in the general population: The association with psychosis expression and risk factors for psychosis

**DOI:** 10.1371/journal.pone.0211914

**Published:** 2019-02-20

**Authors:** E. Schepers, J. van Os, R. Lousberg

**Affiliations:** 1 Department of Psychiatry and Psychology, Maastricht University Medical Centre, Maastricht, The Netherlands; 2 King’s College London, King’s Health Partners, Department of Psychosis Studies, Institute of Psychiatry, London, United Kingdom; 3 Department of Psychiatry, Brain Centre Rudolf Magnus, Utrecht University Medical Centre, Utrecht, The Netherlands; University of La Rioja, SPAIN

## Abstract

**Introduction:**

Positive psychotic experiences are associated with increased rate of white noise speech illusions in patients and their relatives. However, findings have been conflicting to what degree speech illusions are associated with subclinical expression of psychosis in the general population. The aim of this study was to investigate the link between speech illusions and positive psychotic experiences in a general population sample. In addition, the hypothesis that speech illusions are on the pathway from known risk factors for psychosis (childhood adversity and recent life events) to subthreshold expression of psychosis, was examined.

**Methods:**

In a follow-up design (baseline and 6 months) the association between the number of white noise speech illusions and self-reported psychotic experiences, assessed with the Community Assessment of Psychic Experiences (CAPE), was investigated in a general population sample (n = 112). In addition, associations between speech illusions and childhood adversity and life events, using the Childhood Experiences of Care and Abuse questionnaire and the Social Readjustment Rating Scale, were investigated.

**Results:**

No association was found between the CAPE positive scale and the number of white noise speech illusions. The CAPE positive scale was significantly associated with childhood adversity between 12 and 16 years (B = 0.980 *p* = 0.001) and life events (B = 0.488 *p* = 0.044). The number of speech illusions showed no association with either life events or childhood adversity.

**Conclusion:**

In the nonclinical population, the pathway from risk factors to expression of subclinical psychotic experiences does not involve white noise speech illusions as an intermediate outcome.

## Introduction

Psychotic experiences- in the form of attenuated reality distortion, such as bizarre experiences, perceptual abnormalities and persecutory ideas, as observed in psychotic disorders, are also prevalent in the general population [1]. Psychotic experiences co-occur with other domains of subthreshold psychopathology particularly affective dysregulation [2] and the domain of motivational and cognitive alterations [3, 4]. These findings suggest the existence of a multidimensional ‘extended psychosis phenotype’, encompassing a spectrum of severity [3, 4]. Psychotic experiences show phenomenological continuity, in the same psychopathological domains, with psychotic disorder [5], and display transitions in severity over time (temporal continuity) [6–8]. The so-called ‘psychosis-proneness-persistence-impairment model’ suggests that developmental expression of psychosis may become abnormally persistent, and clinically relevant, depending on the degree of environmental exposure interacting with genetic risk [1]. In addition, it has been suggested that psychosis represents a transdiagnostic spectrum, with manifestation in psychotic disorders, emotional disorders, individuals considered at ‘clinical high-risk’ and the general population [4].

The aetiology of altered perceptions and ideation in the context of psychosis remains largely unknown. Theoretical accounts of the cognitive mechanism of hallucinations suggest that normal perception can be viewed as (sensory) bottom-up information combined with top-down information originating from prior knowledge based on previous experience [9–11]. A relatively higher priority to top-down factors compared to bottom-up factors may contribute to the formation of hallucinations [9, 12].

Unlike hallucinations, illusions are distorted interpretations of a 'real' existing stimulus [13]. A number of experimental illusion studies have used an experimental paradigm to examine the tendency to hear voices in white noise (defined as speech illusions), which may operate on top-down processing. The results suggest that the number of speech illusions reflects individual differences in psychosis liability. Thus, Hoffman and colleagues suggested that speech illusions signal an increased risk for transition to a psychotic disorder in a prodromal population [14]. Subsequent research suggested that a White Noise experiment, first reported by Galdos et al. [15] and replicated by Catalan and colleagues [16], can be used to prime and elicit auditory illusions as an index of psychosis liability.

However, there is inconsistency to what degree subclinical expression of psychosis in the general population may be associated with speech illusions generated in the White Noise Task. Both the Structured Interview for Schizotypy-Revised (SIS-R) [17] and the self-report Community Assessment of Psychic Experiences (CAPE) [18, 19] are designed to measure positive psychotic experiences in the general population. Whereas Galdos and colleagues found evidence for an association between any speech illusion and SIS-R positive schizotypy in well controls (odds ratio 2.4) [15], Catalan and colleagues failed to demonstrate an association between speech illusions and either SIS-R positive schizotypy (OR 1.1) or the CAPE positive scale (OR 1.4) [16]. Recently, novel analyses of the expanded sample of the study of Catalan and colleagues showed a significant association between positive schizotypy and speech illusions [OR 4.1]. However, after adjusting for age, sex and WAIS-III, the association was no longer significant and appeared to be mediated to a large extent by cognitive ability [20]. Pries et al. replicated the various statistical methodologies of Catalan and Galdos in a general population twin sample. The results revealed that perception of white noise speech illusion was not associated with either schizotypy (positive or negative) or CAPE score, independent of the methodological strategy used [21]. In a general population cohort of pre-adolescent children, speech illusions were only associated with hallucinations (last month) and negative affect (last month and lifetime) when focussing on affectively salient speech illusions [22].

Given these inconclusive findings, we decided to conduct a new study, not only to reinvestigate the relationship between the number of speech illusions and the CAPE positive scale, but also to examine to what degree experimental (white noise task) and observational measures (CAPE positive scale) of psychosis showed the same pattern of association with known risk factors for psychosis such as childhood trauma [23, 24] and life events [25], which would be compatible with the hypothesis of speech illusions lying on the causal pathway between psychosis risk factors, such as childhood trauma, and psychosis outcome. Alternatively, childhood trauma and life events may have a moderating influence, strengthening the association between speech illusions and psychotic experiences. We thus hypothesized i) a positive association between CAPE and speech illusions; ii) a stronger association between the CAPE positive scale and white noise speech illusions if there was additional evidence for childhood adversity or life events; iii) significant associations between speech illusions and psychosis risk factors of childhood trauma and life events; and iv) positive associations between CAPE and psychosis risk factors.

## Materials and methods

### Participants

One-hundred and twelve persons participated, of whom 95 attended the 6-month follow up assessment. Recruitment of participants was carried out by distributing flyers at public places in Maastricht (population: 120.000). Persons, willing to participate in scientific research about development of (mental) health problems in the general population, were asked to contact the research assistant. Exclusion criteria were the use of antipsychotic, anti-epileptic, antidepressant or anxiolytic medications during the past year and structural excessive use of alcohol (more than 5 units per day). Participants were asked to avoid the use of alcohol the day before the experiment as well as caffeine-containing beverages three hours before the experiment. Participants with hearing problems were excluded. Compensation for participating in both assessments was 50€.

### Ethics statement

The study was conducted according to the principles of the Declaration of Helsinki. The medical ethics committee at Maastricht University Medical Centre approved the study (NL40284.068.12/METC 12-3-015). Written informed consent was given by the participants before the start of the experiment.

### Instruments

#### CAPE

The Community Assessment of Psychic Experiences [18, 19] was used to measure subclinical positive, negative and depressive symptoms. Participants rated both frequency (0 = never to 3 = nearly always) and distress (0 = not distressed to 3 = very distressed) of psychotic experiences. The sum of the frequency scale and the mean of the distress scale was calculated for each domain of psychotic experiences (positive, negative and depressive). In the analysis, only the frequency measure of the positive symptom domain was used. The CAPE has been shown to possess good reliability and validity in general population samples [18].

#### Social Readjustment Rating Scale

A shortened version (14 life events) of the Social Readjustment Rating Scale was used to measure stressful life events [26]. At baseline, individuals rated the presence of life events experienced during the past year. Six months later, at follow up, participants were asked whether they had experienced new events over the follow-up period. The sum of life events rated at baseline and at follow up were calculated. The internal consistency was adequate (Cronbach’s α = 0.622).

#### Childhood adversity

Childhood Adversity (CA) was measured with a questionnaire developed within the context of the FP7 EU-GEI project (European network of national schizophrenia networks studying Gene-Environment Interactions) [27]. Participants were asked to fill in the Childhood Experiences of Care and Abuse questionnaire during baseline assessment. The questionnaire consists of 15 questions on adverse childhood events like presence of financial problems, occurrence of sexual abuse and divorce of parents. Two categories were distinguished: adverse life events between 0 and 11 years (early childhood), and events between 12 and 16 years (adolescence). The sum score of each category was calculated, ranging from 0 to 15. Cronbach’s α was 0.690 for childhood adversity between 0 and 11 years and 0.642 for childhood adversity between 12 and 16 years.

#### Educational level

Highest educational level was assessed and classified according to the Dutch school system (1 = primary education, 2 = pre-vocational secondary education, 3 = senior secondary vocational education and training, 4 = senior general secondary education, 5 = pre-university secondary education, 6 = higher professional education and 7 = university). A dichotomized 0–1 dummy-variable was calculated, 0 indicating low to average educational level (category 1 to 3), and 1 indicating higher educational level (category 4 to 7).

#### The white noise task

The experiment was conducted at baseline and at 6-month follow-up. The white noise task was administered on both occasions and was carried out, for each individual separately, by a master-level research assistant with 5 years of experience. Before the start of the experiment, participants were instructed to listen to sound fragments and to rate the content of the fragments. If there were uncertainties about the instructions, participants could discuss their questions with the research assistant. During the task, each participant was exposed to 75 sound fragments: 25 fragments containing white noise; 25 fragments of white noise mixed with barely audible speech and 25 fragments of white noise mixed with clearly audible speech with a positive, negative or neutral affective context. For example: 'Sport is good for health', 'I think it is going to rain today' or 'Madrid is the capital of Spain'. The presentation of the 75 fragments was in random order and the (clearly and barely audible) sentences were of the same male voice. The fragments were binaurally presented through a wire headset (Plantronics) and the task was carried out in a soundproof room. After the ending of each fragment, participants were asked to press a button on a keyboard in front of them indicating five options: 1: endorsed hearing speech with positive content, 2: endorsed hearing speech with negative content, 3: endorsed hearing speech with neutral content, 4: no speech heard, and 5: endorsed hearing speech but uncertain whether it was positive, negative or neutral. The protocol was guided by the software package 'Presentation' (Version 13.0, Neurobehavioral Systems, Inc.). The word "Listen!" was displayed on the computer screen in front of the participant during the fragment. After a fragment, the five answering options were shown. A new fragment started after an option had been selected by a push on the button. Response time was calculated as the time (in ms) between the end of a fragment and a button push. The total duration of the task varied, since the response time varied per participant per sound fragment. The average duration of the task was 8.9 minutes. In line with the definition proposed by Catalan and colleagues [16], a speech illusion was defined as a white noise only fragment in which any speech was heard (option 1,2,3 or 5). As participants were exposed to 75 fragments, of which 25 contained 'pure' white noise, the maximum number of speech illusions per measurement occasion was 25.

### Statistical analyses

First, the association between the CAPE positive scale and speech illusions was investigated. Number of speech illusions as dependent variable was treated both as continuous and dichotomized variable. The dichotomized score was defined in two ways: as a cut-off value of ≥ 1, conform the methodology proposed by Galdos et al [15], as well as a cut-off value of ≥ 2, as proposed by Catalan and colleagues [16]. Poisson mixed-effects regression models were applied to the non-normally distributed count variable, and binary logistic mixed-effects regression models for the dichotomous variables. As the data were hierarchically organised (measurement occasions nested within individuals), random intercepts for individuals were added to the models. First, models were run with the CAPE positive scale as the predictor of main interest. Second, separate models were executed with speech illusion explained by the interaction between the CAPE positive scale and (i) childhood adversity between 0 and 11 years, (ii) childhood adversity between 12 and 16 years and (iii) life events one year before baseline. Analyses were corrected for age, sex and educational level. Similarly, the Poisson mixed-effects regression model and binary logistic mixed-effect regression models were run with respectively continuous and dichotomised speech illusion as the dependent variable, explained by 4 predictor variables: (i) childhood adversity between 0 and 11 years; (ii) childhood adversity between 12 and 16 years; (iii) life events experienced one year before baseline and (iv) life events between baseline and follow-up. All models were corrected for age, sex and educational level. Finally, linear regression models were run with the CAPE positive scale as dependent variable explained by childhood adversity (between 0 and 11 and between 12 and 16 years) and life events. Since the CAPE positive scale was administered once (at baseline), only life events experienced 1 year before baseline were incorporated in the model, corrected for age sex and educational level. Associations were significant when two-sided p-value was < 0.05. SPSS-version 24 was used as the statistical software package.

## Results

### Participants

One-hundred and twelve participants (age: mean = 39.4; SD = 17.5; sex: 39 men, 73 women) were included at baseline. At 6-month follow up, 95 participants (age: mean = 40.8; SD = 17.3; sex: 34 men, 61 women) were assessed again. Loss-to-follow up analyses were carried out to check for differential attrition. There was no evidence for differential attrition with respect to the rate of speech illusions (*p* = 0.239), age (*p* = 0.053) and sex (*p* = 0.611). Table 1 reports the overview of the demographic variables for baseline and six month follow-up. The median of number of speech illusions at both baseline and follow-up was 1 (Table 2). In total, 461 speech illusions were observed at both measurements. Only two speech illusions (0.4%) were judged as having an affective connotation (one positive and one negative).

**Table 1:** Summary of selected demographic variables and covariates.

	Baseline (n = 112) mean (SD)	Follow-up (n = 95) mean (SD)
Age	39.4 (17.5)	40.8 (17.3)
Female	65.2% (n = 73)	64.2% (n = 61)
Male	34.8% (n = 39)	35.8% (n = 34)
Educational level		
Level 1	5.4% (n = 6)	4.2% (n = 4)
Level 2	8.9% (n = 10)	9.5% (n = 9)
Level 3	17.9% (n = 20)	17.9% (n = 17)
Level 4	5.4% (n = 6)	5.3% (n = 5)
Level 5	28.6% (n = 32)	27.4% (n = 26)
Level 6	18.8% (n = 21)	20.0% (n = 19)
Level 7	15.2% (n = 17)	15.8% (n = 15)
CAPE scale		
Positive dimension	4.6 (4.1)	4.8 (4.1)
Negative dimension	8.0 (5.4)	8.2 (5.7)
Depressive dimension	4.9 (3.2)	5.0 (3.4)
Total number of life events	2.0 (1.5)	1.9 (1.6)
Childhood adversity		
Between 0 and 11	1.7 (1.9)	1.6 (1.8)
Between 11 and 18	1.6 (1.8)	1.6 (1.8)

SD = standard deviation.

**Table 2:** White noise speech illusions during baseline and follow-up.

	Baseline (n = 112)	Follow-up (n = 95)
Total number of SIs	249	212
Percentage of SIs	8.9	8.9
Median number of SIs per participant	1	1

SI = speech illusion

### Prediction models of speech illusions

No association between the CAPE-positive scale and white noise speech illusions could be demonstrated, neither with speech illusions as a continuous variable (B = 0.011, 95% CI -0.053–0.074, *p* = 0.742); nor with a dichotomized variable (cut-off ≥1 B = 0.030, 95% CI -0.049–0.109, *p* = 0.454, cut-off ≥2 B = 0.018, 95% CI -0.067–0.104, *p* = 0.671). There was no evidence that the association between the CAPE-positive scale and white noise speech illusions was moderated by childhood adversity or life events (Table 3). In addition, white noise speech illusions (both as a continuous and as dichotomized variable) were not associated with either childhood adversity or life events (Table 4).

**Table 3:** Interaction between CAPE positive scale and childhood trauma or life events.

	SI; continuous		SI; cut-off ≥2		SI; cut-off ≥1	
	B-coefficient (95% CI)	*p*	B-coefficient (95% CI)	*p*	B-coefficient (95% CI)	*p*
CAPE x Child abuse 0 and 11 years	0.012 (-0.022–0.046)	0.483	0.012 (-0.033–0.056)	0.606	0.025 (-0.019–0.068)	0.265
CAPE x Child abuse 12 and 16 years	0.006 (-0.023–0.036)	0.662	0.006 (-0.035–0.046)	0.781	0.011 (-0.028–0.050)	0.592
CAPE x Life events before baseline	-0.015 (-0.060–0.029)	0.493	-0.029 (-0.089–0.031)	0.346	-0.002 (-0.056–0.052)	0.941

SI = speech illusion

B-coefficient = non-standardized regression coefficient

95% CI = 95% confidence interval

*p* = *p*-value

**Table 4:** Main effect of childhood trauma and life events on white noise speech illusions.

	SI; continuous		SI; cut-off ≥2		SI; cut-off ≥1	
	B-coefficient (95% CI)	*p*	B-coefficient (95% CI)	*p*	B-coefficient (95% CI)	*p*
Child abuse 0 and 11 years	-0.078 (-0.333–0.178)	0.547	-0.117 (-0.493–0.259)	0.537	-0.125 (-0.505–0.255)	0.515
Child abuse 12 and 16 years	0.092 (-0.156–0.340)	0.463	0.209 (-0.156–0.573)	0.258	0.319 (-0.084–0.721)	0.119
Life events before baseline	0.078 (-0.130–0.287)	0.458	0.098 (-0.202–0.399)	0.518	0.010 (-0.290–0.310)	0.946
Life events before follow up	0.141 (-0.229–0.512)	0.450	0.272 (-0.291–0.834)	0.339	-0.063 (-0.632–0.506)	0.826

SI = speech illusion.

B-coefficient = non-standardized regression coefficient

95% CI = 95% confidence interval

*p* = *p*-value

### Prediction models of the CAPE-positive scale

In the linear regression model correcting for age sex and educational level (R-square 0.217), an association was found between the CAPE-positive scale and childhood adversity between 12 and 16 years (B = 0.980, 95% CI 0.389–1.562, *p* = 0.001) and life events one year before baseline (B = 0.488, 95% CI 0.013–0.963, *p* = 0.044). There was no association with childhood adversity between 0 and 11 years (B = -0.210, 95% CI -0.764–0.343 *p* = 0.453).

## Discussion

This study investigated hypothesized associations between white noise speech illusions, psychosis expression and risk factors for psychosis. The initial hypotheses could not be confirmed by the results of the present study. Thus, the analyses demonstrated that: (i) speech illusions were not associated with the CAPE positive scale; (ii) the association between speech illusions and the CAPE positive scale was not moderated by childhood adversity or life events; (iii) speech illusions were not associated with childhood adversity or life events and (iv) the CAPE positive scale was associated with childhood adversity and life events. These findings therefore are not compatible with the hypothesis that early risk factors impact on psychosis expression via speech illusions, or that environmental risks moderate the association between speech illusions and psychotic experiences.

Previous research is inconclusive with respect to the association between speech illusions and observational measures for psychotic experiences (CAPE positive scale and the SIS-R) [15, 16, 21, 22]. In line with the results of Catalan and colleagues and Pries et al., the current study showed no significant association between the CAPE positive scale and the number of speech illusions. This finding might be explained by the theory that observational measures of psychosis and the white noise task focus on different mechanisms underlying psychosis liability in the nonclinical population. The CAPE positive scale mainly indexes alterations in psychosis ideation, whereas the white noise task focusses on alterations in perception. Also, the CAPE positive scale may reflect trait psychosis proneness whereas speech illusions may reflect state variation.

Importantly, the present study focussed on the association between the white noise task and subclinical expression of positive psychotic experiences in the nonclinical population. In the clinical population, imbalance between top-down and bottom-up processing may mediate psychotic symptoms. However, the current findings suggest that in the nonclinical population, altered processing of sensory information is unrelated to the expression of subclinical symptoms. In other words, alteration in perception will not always induce alteration in ideation. Clinical psychosis may reflect the state where alterations in perceptions give rise to delusional explanations [28]. Further research is required to support this theory.

This study is not without shortcomings. Earlier research showed that the association between speech illusions and interview-based measures of positive schizotypy appeared to be mediated by other variables including cognitive ability. Therefore, analyses were corrected for educational level. It might have been more precise, however, to examine cognitive ability with neuropsychological testing. Given the null-finding, however, this lack of sensitivity of the cognitive measure is unlikely to have biased the findings. A second point is related to the choice to include a non-clinical sample. A comparison of effects with a clinical population would have been helpful to better understand possible mediating mechanisms associated with speech illusion on the pathway form risk to psychosis expression. Further study is required to examine this issue.

Finally, no association between white noise speech illusions and expression of subclinical psychotic experiences could be demonstrated in the non-clinical population, suggesting this association is a marker of illness rather than risk. Further research is required, particularly research focussing in non-clinical but at-risk populations such as siblings of patients with a psychotic disorder, who may have higher levels of speech illusions [15].

## Supporting information

S1 File Dataset. (XLSX)
